# Evaluation of dnDSA risk stratification using the updated PIRCHE-T2 model in two kidney transplant cohorts

**DOI:** 10.3389/fimmu.2026.1809989

**Published:** 2026-04-20

**Authors:** Yuan Tian, Matthias Niemann, Benedict M. Matern, Lukas Frischknecht, Anna Mallone, Fabian Rössler, Thomas Schachtner, Stefan Schaub, Jakob Nilsson

**Affiliations:** 1 Department of Immunology, University Hospital Zurich (USZ), Zurich, Switzerland; 2 Research and Development, PIRCHE AG, Berlin, Germany; 3 Department of Nephrology and Medical Intensive Care, Charité Universitätsmedizin Berlin, Berlin, Germany; 4 Center for Translational Immunology, University Medical Center Utrecht, Utrecht, Netherlands; 5 Department of Surgery and Transplantation, University Hospital Zurich, Zurich, Switzerland; 6 Division of Nephrology, University Hospital Zurich, Zurich, Switzerland; 7 Clinic for Transplantation Immunology and Nephrology, University Hospital Basel, Basel, Switzerland

**Keywords:** alloimmunity, donor specific antibodies, kidney transplantation, PIRCHE, risk stratification

## Abstract

**Background:**

Development of *de novo* donor-specific antibodies (dnDSA) remains a key risk factor for antibody-mediated rejection and graft loss in kidney transplantation. The PIRCHE-T2 model estimates immunogenicity by predicting donor-derived HLA peptides presented via the recipient’s HLA class II molecules. A recent update to the model integrates a new neural network-based peptide-binding predictor “Frost”, which requires further testing of clinical performance.

**Methods:**

We compared the predictive performance of the previous and updated PIRCHE-T2 models in two independent kidney transplant cohorts from Zurich (n = 1194) and Basel (n = 387). PIRCHE-T2 scores were assessed at total and locus-specific levels and analyzed in relation to dnDSA incidence using ROC curves, Kaplan-Meier, and Cox regression models.

**Results:**

The updated PIRCHE-T2 model generated lower and more condensed scores but improved dnDSA risk stratification across both cohorts. Higher scores remained associated with increased dnDSA risk. Notable improvements were observed for HLA-C scores. HLA-DQ also showed enhanced performance in one-mismatch subgroups and Cox models, while HLA-A improvements were primarily seen in the Basel cohort. Results for other loci remained similar between models, although HLA-DRB1 showed cohort-specific variation, highlighting the need for context-specific threshold refinement.

**Conclusion:**

Our findings demonstrate that the updated PIRCHE-T2 model refines immunological risk stratification in kidney transplantation, offering improved performance for certain loci and patient subgroups. Its application may support more precise donor selection and individualized immunological assessment. Given observed cohort-specific differences, future work should focus on optimizing thresholds and validating the model across diverse populations to ensure broader clinical applicability.

## Introduction

The presence of pre-transplant donor-specific antibodies (DSA) or the development of *de novo* donor-specific antibodies (dnDSA) remains a central challenge in kidney transplantation, as it is closely linked to the increased risk of antibody-mediated rejection (ABMR) and poor long-term graft outcomes (1–4). ABMR is thought to be primarily driven by a T cell-dependent alloimmune response against mismatched donor human leukocyte antigen (HLA) proteins, which serve as key immunological targets. The presence of dnDSA not only mediates graft injury, but also often necessitates more intensive immunosuppressive treatment, which further increases the risk of infection (5, 6). In cases of graft failure due to ABMR, re-transplantation can be hindered by broad sensitization and high calculated panel reactive antibody (cPRA) levels, significantly limiting donor availability (7). These challenges highlight the need to prevent dnDSA formation and subsequent development of ABMR to help protect kidney grafts and improve long-term outcomes for transplanted patients.

Although HLA mismatches are known to increase the risk of dnDSA formation and graft loss, not all mismatches carry equal immunological weight. Traditional mismatch counting does not account for the qualitative immunogenic potential of specific donor-recipient combinations. To address this limitation, the Predicted Indirectly Recognizable HLA Epitopes (PIRCHE-T2) model was developed. This model estimates the number of mismatched donor HLA-derived peptides that can be presented by the recipient’s HLA-DRB1 molecules, thereby offering a peptide-level metric of indirect alloantigen recognition potential. Prior studies, including our own, have demonstrated a robust association between higher PIRCHE-T2 scores and the subsequent development of dnDSA and graft injury (8–14).

Recently, the PIRCHE-T2 model was updated with a newly developed HLA peptide-binding predictor, “Frost,” based on neural network models. This enhancement aims to improve the prediction of peptide-MHC binding by more precisely modeling the highly polymorphic HLA-DRB1 binding groove. However, it remains unclear how this update affects the performance of the PIRCHE-T2 model in predicting dnDSA development risk in kidney transplantation compared to the earlier model.

In this study, we analyzed two large, well-characterized cohorts of kidney transplant recipients from the University Hospital Zurich (n = 1194) and the University Hospital Basel (n = 387) to evaluate and compare the predictive performance of the original and updated PIRCHE-T2 scoring models. Both cohorts are part of previously published studies, with earlier analyzes based on a subset of the current dataset. Prior findings highlighted key immunogenetic and clinical contributors to alloimmunity, including associations between PIRCHE-T2 scores and dnDSA formation, as well as the added predictive value of combining molecular mismatch approaches such as eplet mismatches and immunogenic eplets in risk stratification (11, 15). These findings demonstrate the clinical relevance and consistency of both datasets. Here, we investigated associations between PIRCHE-T2 scores and dnDSA development at total and HLA locus-specific levels, and assessed the consistency of score distributions, risk thresholds, and time-dependent dnDSA outcomes across both cohorts. By integrating data from multiple transplant programs, our aim was to assess the performance of the updated PIRCHE-T2 model and examine its potential contribution to improving pre-transplant immunological risk stratification in kidney transplantation.

## Methods

### Patient population

The Zurich cohort is built on a previously published cohort of kidney transplant recipients at the University Hospital Zürich (USZ), as previously described (11). The original cohort included patients who underwent kidney transplantation between January 2008 and March 2024 and were routinely monitored for *de novo* donor-specific antibody (dnDSA) development post-transplantation. For the current study, we used the same dataset with “False” dnDSA patients excluded, as defined below, resulting in the final cohort comprising 1194 patients, which includes detailed immunological information such as recipient and donor HLA typing, transplantation date, previous and new version PIRCHE-T2 scores (HLA-A, B, C, DRB1, and DQB1), follow-up duration, and data on dnDSA development. The study was approved by the local Ethical Committee in Zurich (BASEC 2018-01182).

The Basel cohort included kidney transplant recipients at the University Hospital Basel between January 2014 and August 2022. A total of 387 standard risk patients were included, all with complete HLA typing and dnDSA follow-up data (15). A similar set of clinical and immunological variables, including HLA typing and dnDSA status, were collected as in the Zurich cohort. The study of the Basel cohort was approved by the local ethics committee (EKNZ 2023-01992).

### HLA typing and dnDSA evaluation

HLA typing and anti-HLA antibody analysis were performed as previously described (11, 15). HLA typing was conducted using SSO, SSP, or NGS-based methods. Low-resolution typing was used in this study. Post-transplant anti-HLA antibody screening followed institutional protocols using Luminex single-antigen bead (SAB) assays, with a positivity cut-off at 500 MFI. Routine testing was performed at 1, 3, 6, 12, 18, and 24 months post-transplant, and yearly thereafter. Additional testing was performed as clinically indicated. dnDSA were identified via an automated virtual crossmatch (vXM) algorithm based on antigen-level HLA typing and SAB profiles.

Each detected dnDSA was individually reviewed in a blinded manner by a transplantation immunology specialist, and designated according to our previously published clinical system (11, 15, 16). Donor specificity, antibody epitope binding pattern (including alpha-chain specificity for HLA-DQ and HLA-DP), and potential unspecific reactivity (including lot-specific patterns and self-HLA reactivity) were systematically assessed. In the Zurich cohort, dnDSA were then categorized into three groups: “True” dnDSA, showing clear donor-specific reactivity without evidence of unspecific binding; “Possible” dnDSA, where specificity could not be definitively confirmed or excluded; “False” dnDSA, showing no donor specificity or clear non-specific reactivity. For the purposes of this study, “True” and “Possible” dnDSA were considered as dnDSA, while False dnDSA were excluded. In contrast, the Basel cohort only included dnDSA classified as “True”, as this was how the data were originally recorded in that center’s database.

Patients with pre-existing DSA prior to transplantation were only considered dnDSA-positive if new antibody specificities developed post-transplant.

### PIRCHE-T2 score calculation and threshold definition for original and updated models

The PIRCHE-T2 model estimates the number of indirectly recognizable donor-derived HLA peptides presented by the recipient’s HLA class II molecules, focusing primarily on HLA-DRB1. In version 3, it uses the NetMHCIIpan 3.2 tool to predict peptide binding affinities, counting unique donor-derived 15-mer peptides with predicted binding affinities below 1000 nM as potential T-cell epitopes (17, 18). Scores were calculated separately for each HLA locus, HLA-A, HLA-B, HLA-C, HLA-DRB1, and HLA-DQB1, with class I scores derived by summing HLA-A, HLA-B, and HLA-C scores, and class II scores combining HLA-DRB1 and HLA-DQB1. The total PIRCHE-T2 score represented the number of unique peptide-HLA tuples derived from all considered loci.

The key difference in the new PIRCHE version 4 is the introduction of a new peptide binding predictor referred to as Frost (as introduced in (18, 19)). This binding predictor, which is based on an ensemble of 128 artificial neural networks (ANNs), predicts a ranked binding strength of donor-derived peptides by encoding features of the highly polymorphic HLA-DRB1 binding groove. These predictions give indications of binding preferences of the distinct HLA-DRB1 presenters, and facilitate the downstream prediction of which donor-derived HLA peptides are likely to result in transplant immunogenicity. Incorporating the previously suggested binding affinity ranking and most recent Immune Epitope DataBase (IEDB) training data, this enhancement aims to improve the precision of immunogenicity assessments in the context of organ transplantation.

The previously established PIRCHE-T2 score cut-off of 15 per locus, based on our earlier work (11), was applied consistently in both cohorts. To adapt to the generally lower PIRCHE-T2 scores in version 4, new cut-offs were defined to yield patient group sizes comparable to those generated by the original threshold. For example, for HLA-A, thresholds of approximately 12 in the Basel cohort and 13 in the Zurich cohort resulted in a similar distribution of patients in the low-score group as with the original cut-off of 15.

### Statistical methods

Several statistical tests were applied to assess significance in this study. These included Kaplan-Meier analysis with the log-rank test for comparing the incidence of dnDSA between groups, and Cox proportional hazards regression for multivariable time-to-event analysis. Differences in PIRCHE-T2 scores were tested using Wilcoxon tests: the rank-sum test for comparisons between different patient groups, and the signed-rank test for paired comparisons within the same patients.

All analyzes were performed in R (version 4.2.4). Data were imported and processed using readxl (1.4.3) and dplyr (1.1.2). Donut/pie charts were generated with a base R function. Violin plots were generated with ggplot2 (3.4.2). Survival analyzes, including Kaplan-Meier curves, Cox proportional hazards models, and log-rank tests, were performed using survival (3.5.5) and visualized with survminer (0.4.9). Group comparisons of continuous variables were conducted using Student’s t-test (base R). Heatmaps were created with ComplexHeatmap (2.14.0) (20), which relies on circlize (0.4.16) for layout and color mapping (21).

## Results

### Distribution of dnDSA and PIRCHE-T2 model comparison

**Figure 1:**
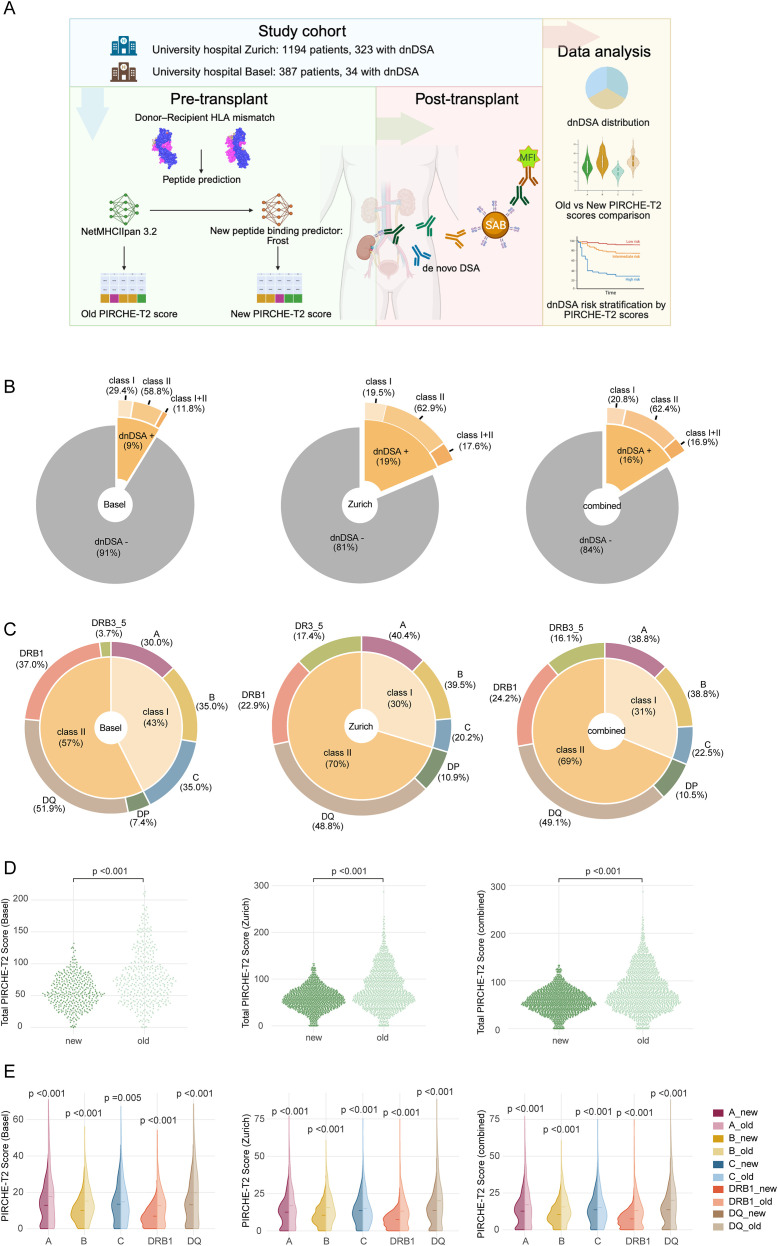
Overview of study cohorts, dnDSA distribution, and PIRCHE-T2 score comparisons. **(A)** Schematic illustration of the study workflow. Created in BioRender. Nilsson, J. (2026) https://BioRender.com/d7od0jl. **(B)** Proportion of dnDSA-positive patients in each cohort. **(C)** Distribution of dnDSA targets across HLA loci, grouped into class I and class II responses. **(D)** Comparison of total PIRCHE-T2 scores generated by the old and updated models. **(E)** Locus-specific comparison of PIRCHE-T2 scores across scoring versions for HLA-A, -B, -C, -DRB1, -DQ.

A visual overview of the study cohort and the PIRCHE-T2 scoring workflow is shown, including cohort information, HLA mismatch processing, peptide prediction using both models, and the resulting dnDSA assessment (**Figure 1A**). Median follow-up was 4.53 years (IQR 1.96–8.19) in the Zurich cohort and 3.94 years (IQR 1.99–5.75) in the Basel cohort. The distribution of dnDSA events, targeted loci, and PIRCHE-T2 score comparisons across the Basel, Zurich, and combined cohorts, respectively, are summarized alongside (**Figures 1B–E**). dnDSA development post-transplant was more frequently observed in the Zurich cohort (19%) compared to Basel (9%), with a combined prevalence of 16% (**Figure 1B**). This discrepancy likely reflects the inclusion of both “True” and “Possible” dnDSA cases in the Zurich cohort (see Methods section), whereas only “True” dnDSA were included in the Basel cohort.

Across all cohorts, the majority of dnDSA targeted class II HLA, particularly DQ and DRB1, while class I dnDSA remained less common (30-43%). Class II responses accounted for about 60% of all dnDSA cases, with similar distribution across both centers. Among class II targets, DQ-specific antibodies predominated, followed by DRB1, whereas donor-specific antibodies against DP were relatively infrequent (**Figure 1C**).

Compared to the previous version, the updated PIRCHE-T2 model produced lower and more condensed total scores (p < 0.001), indicating a systematic shift in score distribution (**Figure 1D**). This trend was consistent across both cohorts. When comparing individual HLA loci, all showed significantly reduced scores under the updated model (p < 0.001), with the most pronounced decrease observed at DRB1 and DQ (**Figure 1E**).

### Comparison of PIRCHE-T2 models in relation to dnDSA development

**Figure 2:**
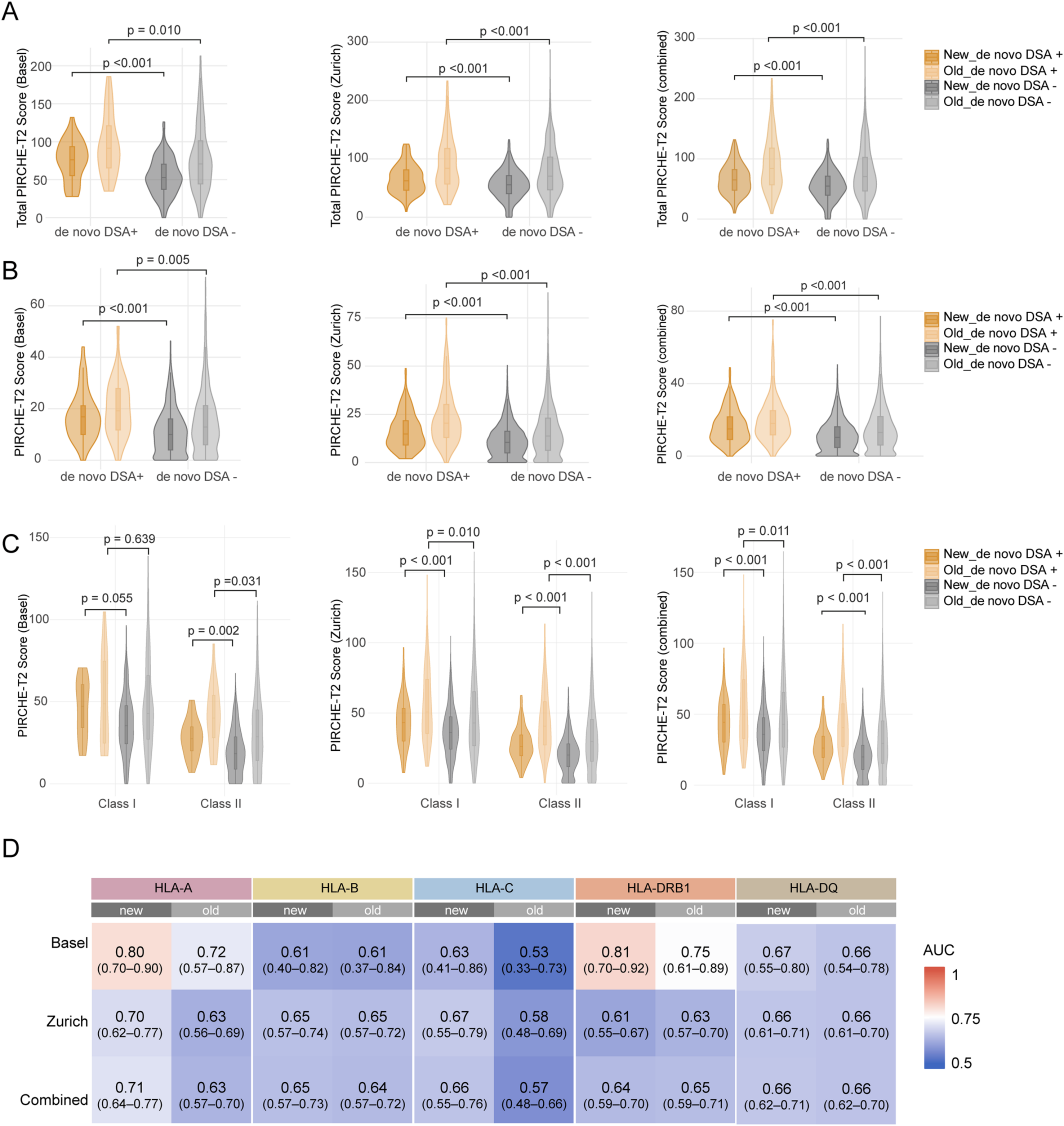
Comparison of PIRCHE-T2 scores between dnDSA-positive and -negative patients. **(A)** Total PIRCHE-T2 scores stratified by dnDSA status. **(B)** Event-level PIRCHE-T2 scores for dnDSA and non-dnDSA events, shown for descriptive purposes only; multiple events per patient may be represented. **(C)** Class-specific (class I and class II) PIRCHE-T2 scores between dnDSA groups. **(D)** ROC curve analysis showing AUC values per HLA locus for both the old and updated PIRCHE-T2 models. AUC values are presented with 95% confidence intervals.

PIRCHE-T2 scores were significantly higher among dnDSA-positive patients than those without dnDSA across all cohorts, with improved separation under the new model in the Basel dataset (**Figure 2A**). When scores were broken down by individual dnDSA events (**Figure 2B**), similar patterns were observed: dnDSA-positive patients consistently showed significantly higher scores than dnDSA-negative patients, again with better separation under the updated PIRCHE-T2 in Basel.

These findings remained consistent at the HLA Class level, with Class II PIRCHE-T2 scores being significantly elevated in dnDSA-positive patients across all datasets, with the most pronounced differences seen in the Zurich and combined cohorts (all p < 0.001) (**Figure 2C**). Class I scores reached statistical significance only in Zurich and the combined cohort, with improved separation under the updated model. In the smaller Basel cohort, class I scores did not significantly differ between dnDSA positive and negative groups, but the separation trend improved with the new score.

To further assess the predictive value of the updated PIRCHE-T2 model, we calculated area under the curve (AUC) values for each locus and PIRCHE-T2 model version (**Figure 2D**). Across all cohorts, the updated model showed either a modest improvement or similar performance, with the most notable gains observed at HLA-C (AUC: new 0.63 vs. old 0.53 in Basel; new 0.67 vs. old 0.58 in Zurich) and HLA-A (new 0.80 vs. old 0.72 in Basel; new 0.70 vs. old 0.63 in Zurich). DRB1 showed the highest AUCs in the Basel cohort (0.81 new vs. 0.75 old), while in Zurich, its performance was comparable between models (0.63 old vs. 0.61 new), suggesting a potential center-specific difference. These trends are further supported by the violin plots in **Supplementary Figure 1**, which show significantly higher locus-specific PIRCHE-T2 scores among dnDSA positive patients compared to dnDSA negative ones. The updated model consistently showed stronger separation across most loci.

Overall, these results suggest that the new PIRCHE-T2 model has improved dnDSA prediction, with some variability across loci and cohorts.

### Kaplan–Meier analysis of dnDSA development stratified by PIRCHE-T2 model and HLA locus mismatches

**Figure 3:**
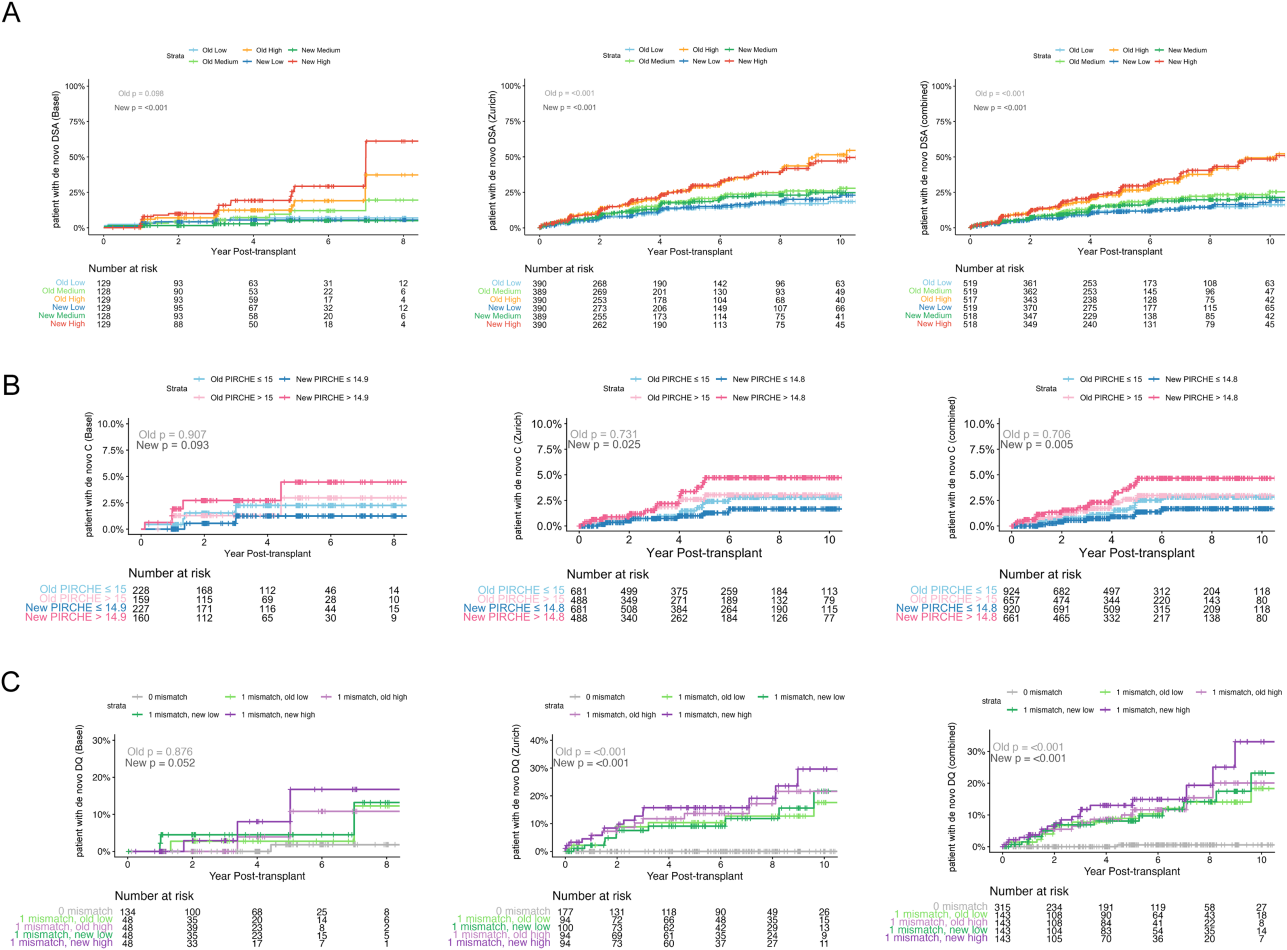
Kaplan–Meier plots of dnDSA incidence stratified by PIRCHE-T2 scores and HLA mismatch patterns. **(A)** Cumulative incidence of dnDSA based on total PIRCHE-T2 tertiles (low, medium, high). **(B)** Cumulative incidence of HLA-C specific dnDSA, stratified by PIRCHE-T2 score using binary thresholds. The original score applied a fixed cutoff of 15; the updated score used a threshold yielding a comparable patient distribution. **(C)** Cumulative incidence of dnDSA in patients with one HLA-DQ mismatch, stratified by DQ PIRCHE-T2 score using binary thresholds. Groups represent the top and bottom quartiles of the score distribution.

To evaluate whether the updated PIRCHE-T2 model maintained or improved its predictive capacity over time, we next performed Kaplan–Meier analyzes to evaluate dnDSA incidence across stratified PIRCHE-T2 score groups in the Basel, Zurich, and combined cohorts. Patients were grouped into tertiles (low, medium, high) based on the total PIRCHE-T2 score distribution (**Figure 3A**), and binary thresholds were applied for locus-specific analysis (**Figures 3B, C**) based on established cutoffs.

In the Basel cohort, the updated total score yielded markedly improved separation between risk groups, particularly identifying high-risk subset with significantly increased dnDSA incidence over time (new p < 0.001). In contrast, the old model showed only a non-significant trend (old p = 0.098). In Zurich and the combined cohorts, both models produced strong risk stratification (all p < 0.001), with the updated score achieving similar separation of dnDSA risk curves (**Figure 3A**).

Focusing on HLA-C associated dnDSA (**Figure 3B**), we observed that the old PIRCHE-T2 model had limited predictive value in our Zurich cohort. The updated model showed significantly better separation between >14.8 and ≤14.8 score groups in Zurich (p = 0.025) and in the combined dataset (p = 0.005), whereas the old model failed to reach statistical significance in any cohort. In Basel, the updated model also showed improved separation of the risk groups (p = 0.093), as compared to the old version (p = 0.907).

To validate the discriminative performance of the predefined cutoffs used for stratification, we performed receiver operating characteristic (ROC) analyzes for HLA-C–associated dnDSA across the Basel, Zurich, and combined cohorts (**Supplementary Figure 7**). Across all cohorts, the updated PIRCHE-T2 model consistently demonstrated improved discrimination compared to the original model, with higher area under the curve (AUC) values. The predefined cutoffs showed balanced sensitivity and specificity, while Youden index–derived thresholds yielded comparable or slightly higher sensitivity at the expense of specificity. These findings support the use of the selected cutoffs for Kaplan–Meier stratification and confirm the improved predictive performance of the updated model.

Similar improvements for other loci are demonstrated in **Supplementary Figure 2**, where the updated model shows clearer separation for HLA-A (panel A) and DRB1 (panel C) in the Basel cohort. However, improvements were not consistent in Zurich and the combined cohorts, where HLA-B and HLA-DRB1 (panels B and C) showed slightly reduced separation with the new model compared to the old.

As shown in our previous analysis, dnDSA risk was most strongly stratified by PIRCHE scores in patients with one mismatch at the HLA-DQ locus, with the highest and lowest score quartiles displaying the greatest separation (11). We further performed a subgroup analysis of patients with one mismatch at the HLA-DQ locus (**Figure 3C**). Again, the updated model provided clearer separation in dnDSA risk across all cohorts, with significant differences observed in Zurich (p < 0.001) and the combined dataset (p < 0.001). While both the old and new model reached statistical significance in these cohorts, the updated model resulted in steeper divergence of risk curves. In the Basel cohort, the old model failed to separate dnDSA risk (p = 0.876), while the updated model showed marked improvement (p = 0.052). Comparable analyzes for other one-mismatch loci are shown in **Supplementary Figure 3**. Notably, for HLA-DRB1 (**Supplementary Figure 3D**), the updated model improved risk separation only in the Basel cohort, while in Zurich its performance slightly declined. For HLA-A (**Supplementary Figure 3A**), improved separation was observed in Basel, with comparable performance in Zurich.

Together, these findings suggest that the updated model enhances the predictive resolution of the PIRCHE-T2 score for dnDSA risk prediction over time, particularly for HLA-C and HLA-DQ (derived from DQB1 typing in the PIRCHE model) mismatches. While improvements were generally observed across cohorts, some effects, such as those for HLA-DRB1, appeared to be cohort-specific, highlighting potential differences in population characteristics or data composition. The updated model may thus better capture risk in patients with intermediate mismatch profiles, though center-specific validation remains important.

### Cox regression-based stratification of dnDSA-free survival by PIRCHE-T2 model

To further assess the time-dependent predictive value of the PIRCHE-T2 score, we performed Cox proportional hazards regression and visualized dnDSA-free survival curves stratified by tertiles of both the previous and updated PIRCHE-T2 model across the Basel, Zurich and combined cohorts.

**Figure 4:**
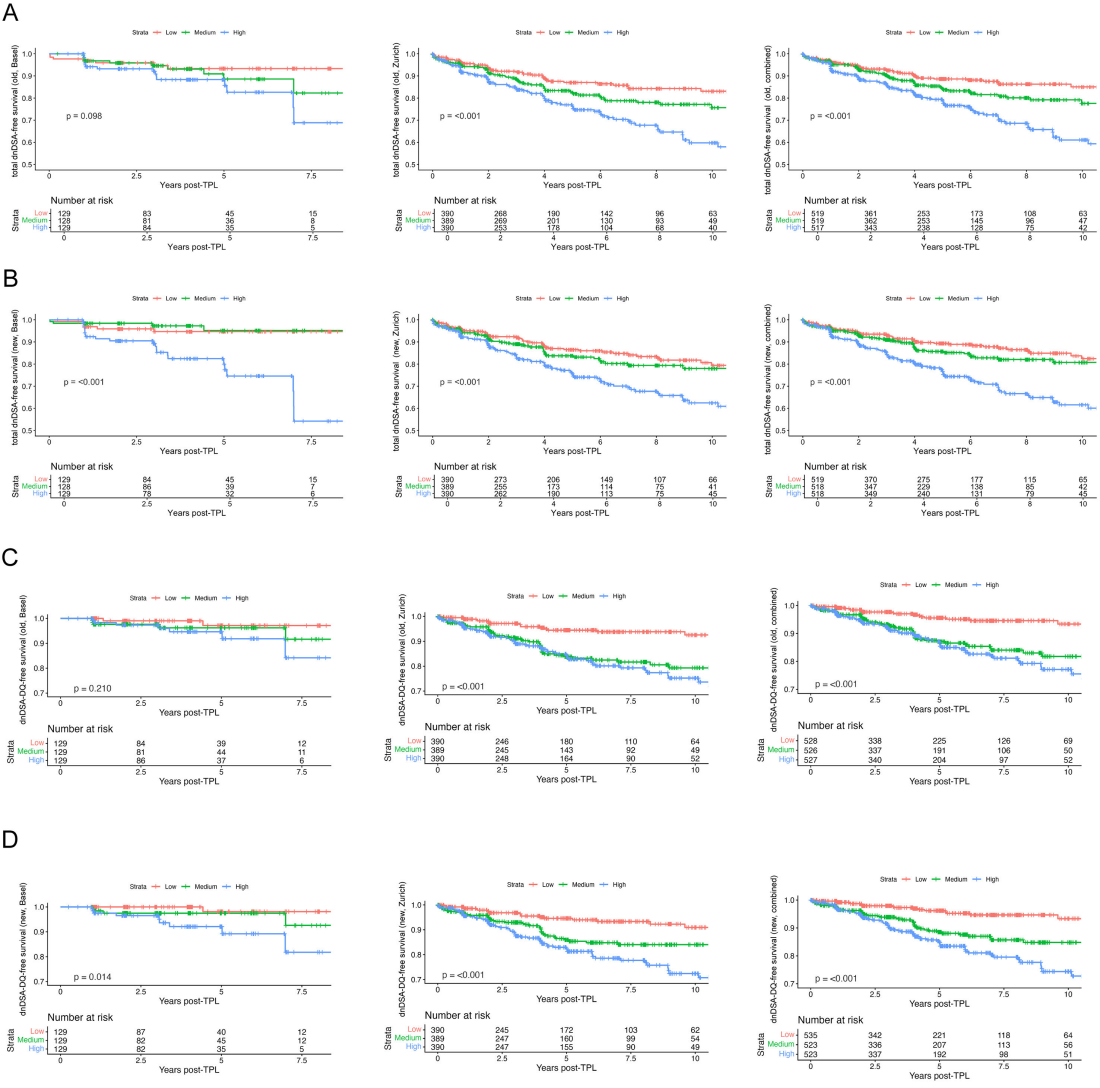
Univariable Cox proportional hazards models showing dnDSA-free survival based on PIRCHE-T2 score stratification. **(A, B)** Cox proportional hazards model of dnDSA-free survival stratified by tertiles of total PIRCHE-T2 scores. A, old PIRCHE scores (Basel: low ≤56.1, medium 56.1-90.2, high >90.2; Zürich: low ≤55.9, medium 55.9-94.4, high >94.4; combined: low ≤56.0, medium 56.0-93.7, high >93.7); **(B)**, new PIRCHE scores (Basel: low ≤42.9, medium 42.9-67.0, high >67.0; Zürich: low ≤47.0, medium 47.0-66.8, high >66.8; combined: low ≤46.4, medium 46.4-67.0, high >67.0). **(C, D)** Cox proportional hazards model of dnDSA-free survival stratified by tertiles of HLA−DQ-specific PIRCHE-T2 scores. C, old PIRCHE scores (Basel: low ≤10.0, medium 10.0-26.0, high >26.0; Zürich: low ≤11.9, medium 11.9-25.3, high >25.3; combined: low ≤11.5, medium 11.5-25.4, high >25.4); D, new PIRCHE scores (Basel: low ≤7.9, medium 7.9-16.5, high >16.5; Zürich: low ≤9.2, medium 9.2-17.0, high >17.0; combined: low ≤9.0, medium 9.0-17.0, high >17.0).

The previous total PIRCHE-T2 model (**Figure 4A**) showed significant separation between risk groups in the Zurich and combined cohorts (both p < 0.001), while the Basel cohort exhibited a non-significant trend (p = 0.098). In contrast, the updated total PIRCHE-T2 model (**Figure 4B**) provided clearer and more consistent stratification across all three cohorts. Although the separation between the low and medium groups became narrower in all three cohorts, the overall analysis remained strongly significant (all p < 0.001).

When focusing on HLA-DQ, the most frequent target of dnDSA in our cohorts, results based on the older DQ score (**Figure 4C**) showed no significant group separation in Basel (p = 0.210), while it performed well in Zurich and the combined cohort (both p < 0.001). Notably, the updated DQ score (**Figure 4D**) improved risk stratification across all cohorts, including Basel (p = 0.014), and showed more distinct separation between the medium and high groups in Zurich and the combined datasets. Additional Cox regression analyzes for other loci are shown in **Supplementary Figures 4**, **5**, comparing the old and new model, respectively. The updated model provided improved risk stratification for HLA-A and HLA-C across all cohorts, while DRB1 showed enhanced separation primarily in Basel. In Zurich, however, the DRB1-specific curves displayed an inverted order between the medium- and high-risk groups, though both remained clearly distinct from the low-risk category (**Supplementary Figure 4D** and **Supplementary Figure 5D**).

To quantify the predictive performance of the total PIRCHE-T2 models, we additionally evaluated hazard ratios (HR) and model discrimination using the concordance index (C-index). Across all cohorts, the updated PIRCHE-T2 model demonstrated consistently higher hazard ratios and improved discrimination compared to the previous model. In the Zurich cohort, the updated model showed a C-index of 0.597 compared to 0.576 for the previous model, with corresponding hazard ratios of 2.398 (95% CI 1.692–3.399) versus 1.797 (95% CI 1.397–2.313). Similarly, in the Basel cohort, model performance improved substantially (C-index 0.669 vs. 0.588; HR 7.361 [95% CI 2.788–19.431] vs. 2.421 [95% CI 1.240–4.724]). In the combined cohort, the updated model also outperformed the previous version (C-index 0.612 vs. 0.578; HR 2.846 [95% CI 2.045–3.960] vs. 1.877 [95% CI 1.481–2.379]). These results further support the improved predictive performance of the updated PIRCHE-T2 model.

In our study, the definition of dnDSA differed between centers, with the Zurich cohort including both “True” and “Possible” dnDSA cases. To assess the impact of this difference, key analyzes were repeated in the Zurich cohort restricted to “True” dnDSA only. These analyzes yielded consistent results across all analyzes, indicating that the inclusion of “Possible” dnDSA did not materially influence the findings (**Supplementary Figure 6**).

Together, these Cox regression-based analyzes, supported by improved hazard ratios and model discrimination, demonstrate the enhanced performance of the updated PIRCHE-T2 model in longitudinal risk prediction, particularly for HLA-DQ directed alloimmune responses.

## Discussion

Our study provides a comprehensive evaluation of the updated PIRCHE-T2 model (version 4), which incorporates the new Frost HLA peptide-binding predictor, in assessing the risk of *de novo* donor-specific antibody (dnDSA) development following kidney transplantation. By analyzing two large, well-characterized cohorts including a total of 1581 kidney transplant recipients with excellent HLA typing data as well as detailed data on dnDSA development post-transplant, we compared the predictive performance of the updated model against the previous version 3. We focused on total and locus-specific scores and the ability to define risk groups as well as 1-mismatch associated risk.

Consistent with prior research, our findings confirm the significant association between higher PIRCHE-T2 scores and increased risk of dnDSA formation (8, 11, 22). Across both cohorts, the new model consistently yielded lower and more condensed scores, likely reflecting refined modeling of the HLA peptide-binding groove. Importantly, this model change did not compromise the predictive value, in fact, in several analyzes, including AUC and Cox regression models, the new PIRCHE-T2 model demonstrated improved or equivalent performance as compared to the previous model. This suggests an improved specificity of the Frost model by exclusion of false-positive T cell epitopes. The most pronounced improvements were seen for HLA-C and HLA-A (both cohorts), with modest gains or comparable performance at other loci.

However, DRB1 in the Basel cohort maintained high predictive value, whereas in Zurich, performance was more variable across analyzes, suggesting potential center-specific variation. While DRB1-specific dnDSA were more frequent in Basel compared to Zurich, this difference alone does not fully explain the observed patterns. Notably, the applied univariable comparisons of PIRCHE-T2 score versions mask potential impact of antibody epitope incompatibility. Higher average scores in the previous PIRCHE version inherently cover more antibody epitope mismatches by chance and thus the model may appear more performant, although it is less specific for its designed purpose of predicting indirect T cell alloreactivity. Similarly, in the Cox regression analysis, dnDSA incidence varied across risk groups. Although high-risk groups typically showed increased risk, in Zurich, the updated DRB1 score yielded similar incidence between the high- and medium-risk categories, while the low-risk group remained clearly separated. In contrast, the Basel cohort showed improved risk stratification with the updated DRB1 score. We plan to explore the underlying causes further, including the potential influence of peptide-binding affinity differences within the cohort, which may help optimize risk prediction and improve individualized pre-transplant immunological assessment.

Longitudinal risk analyzes using Kaplan–Meier and Cox models further supported the enhanced stratification of dnDSA risk using the updated PIRCHE-T2 model. Particularly in patients with one HLA-DQ mismatch, the new model provided clearer separation between risk groups. This was especially evident in the Zurich cohort, where the updated model improved stratification across all three tertiles in the Cox proportional hazards model, whereas the old model only separated the medium- and high-risk groups in this one-mismatch subset. These findings are clinically relevant, as such patients may not be captured by traditional mismatch counting but still carry meaningful alloimmune risk (23).

Importantly, both the Zurich and Basel cohorts were previously published and reflect real-world clinical heterogeneity. The consistent findings across two centers, despite methodological and demographic differences, strengthen the generalizability of our conclusions. At the same time, this heterogeneity highlights the need for context-specific threshold refinement when implementing these scores in clinical decision-making.

Several limitations should be noted. The inconsistent classification of dnDSA, using both “True” and “Possible” cases in Zurich, but only “True” in Basel, may have introduced bias and highlights the need for harmonized definitions in future multicenter studies. To address this, we performed analyzes restricted to “True” dnDSA cases in the Zurich cohort, which yielded comparable results, supporting the robustness of the main findings. Nevertheless, these differences highlight the need for harmonized dnDSA definitions in future multicenter studies. In addition, multivariable adjustment was not performed due to limited availability of key clinical covariates, including immunosuppression regimens and other relevant patient factors across cohorts. As a result, the independent predictive value of the PIRCHE-T2 score beyond established clinical risk factors could not be assessed, and findings should be interpreted accordingly. Furthermore, while dnDSA classification included antibodies against both DQA and DQB chains, the absence of DQA1 typing prevented its incorporation into PIRCHE-T2 analyzes. As DQ immunogenicity is determined by the DQA1–DQB1 heterodimer, restricting the analysis to DQB1-derived peptides likely underestimates the true epitope load and may attenuate DQ-specific associations. While prior studies have demonstrated strong overall correlation between PIRCHE-II scores derived from imputed low-resolution and bona fide high-resolution HLA typing at the population level, as well as similar hazard ratios for dnDSA development, an effect of our reliance on imputed high-resolution typing on risk category assignment for individual patients cannot be excluded (24). This may attenuate effect sizes and reduce the accuracy of individual risk stratification. More broadly, the PIRCHE-T2 model is based solely on T cell epitope prediction and does not account for B cell epitope specificity or linked recognition, both of which are essential for antibody formation treats predicted peptides in an aggregate manner without weighting their individual immunogenicity, and focuses on peptide presentation via HLA-DRB1 without explicitly incorporating other class II loci such as HLA-DQ or HLA-DP. These conceptual limits should be considered when interpreting the scores.

In conclusion, the updated PIRCHE-T2 model shows potential for improved immunological risk stratification in kidney transplantation, particularly for HLA-C-associated responses. These findings support its potential use in pre-transplant donor selection, risk-adapted immunosuppression, and personalized transplant strategies. Although the patient cohorts have been used in previous studies, this analysis represents the first application of the updated PIRCHE-T2 model. Nevertheless, future validation in fully independent or prospectively collected cohorts, as well as studies integrating clinically relevant endpoints such as rejection and graft loss, will be essential to confirm its generalizability and define clinically actionable thresholds.

## Data Availability

The raw data supporting the conclusions of this article will be made available by the authors, without undue reservation.

## Supplementary material

Supplementary Figure 1 Locus-specific PIRCHE-T2 scores stratified by dnDSA status. **(A–C)** Violin plots comparing PIRCHE-T2 scores between dnDSA-positive and -negative patients across individual HLA loci (HLA-A, -B, -C, -DRB1, and -DQB1) within the Basel **(A)**, Zurich **(B)**, and combined **(C)** cohorts. Each panel displays results for both the original (light color) and updated (dark color) PIRCHE-T2 models, illustrating locus-specific differences in predicted immunogenicity by dnDSA status.

Supplementary Figure 2 Kaplan–Meier curves for locus-specific dnDSA development using binary PIRCHE-T2 score thresholds. **(A–D)** Cumulative incidence plots of dnDSA development against HLA-A, -B, -DRB1, and -DQ, respectively, across the Basel (left), Zurich (middle), and combined cohorts (right). For each locus, patients were stratified using binary PIRCHE-T2 score thresholds (original version: ≤15 vs. >15; updated version: adjusted thresholds to match patient group sizes). Both the original (light lines) and updated (dark lines) scores are shown to compare time-dependent dnDSA risk across model versions.

Supplementary Figure 3 Kaplan–Meier curves for dnDSA development in patients with one HLA mismatch per locus. **(A–D)** Cumulative incidence plots of dnDSA development in patients with exactly one mismatch at HLA-A, -B, -C, and -DRB1, respectively, across the Basel (left), Zurich (middle), and combined cohorts (right). Patients were divided using quantile-based thresholds of the old (light lines) and updated (dark lines) PIRCHE-T2 scores, grouped into low (bottom quantile) and high (top quantile) score categories.

Supplementary Figure 4 Cox proportional hazards models for dnDSA-free survival using old PIRCHE-T2 scores. **(A–E)** Cox regression plots showing dnDSA-free survival stratified by tertiles of the old PIRCHE-T2 scores for HLA-A, -B, -C, -DRB1, and -DQB1. Separate analyzes are shown for the Basel (left), Zurich (middle), and combined cohorts (right). Tertile thresholds were derived from the respective score distributions within each cohort.

Supplementary Figure 5 Cox proportional hazards models for dnDSA-free survival using updated PIRCHE-T2 scores. **(A–E)** Cox regression plots showing dnDSA-free time stratified by tertiles of the updated PIRCHE-T2 scores for HLA-A, -B, -C, -DRB1, and -DQB1. Separate panels represent the Basel, Zurich, and combined cohorts. These curves correspond to the analyzes in **Supplementary Figure 4** but use the new PIRCHE-T2 scoring **model** to assess changes in time-dependent predictive value.

Supplementary Figure 6 Analyses restricted to “True” dnDSA cases in the Zurich cohort. **(A)** Proportion of dnDSA-positive patients considering only “True” dnDSA cases. **(B)** Distribution of dnDSA targets across HLA loci, grouped into class I and class II responses. **(C)** Cumulative incidence of dnDSA based on tertiles (low, medium, high) of total PIRCHE-T2 scores. **(D)** Cumulative incidence of HLA-C specific dnDSA stratified by PIRCHE-T2 score using binary thresholds. **(E)** Cumulative incidence of dnDSA in patients with one HLA-DQ mismatch, stratified by DQ PIRCHE-T2 score using top vs. bottom quartiles. **(F–G)** Cox proportional hazards models of dnDSA-free survival stratified by tertiles of total PIRCHE-T2 scores. **(F)** Old PIRCHE scores; **(G)** updated PIRCHE scores. **(H–I)** Cox proportional hazards models of dnDSA-free survival stratified by tertiles of HLA-DQ-specific PIRCHE-T2 scores. **(H)** Old PIRCHE scores; **(I)** updated PIRCHE scores.

Supplementary Figure 7 ROC analysis of HLA-C–specific PIRCHE-T2 scores. **(A–C)** ROC curves for HLA-C specific PIRCHE-T2 scores in the Basel, Zurich, and combined cohorts. The original (light blue) and updated (dark blue) models are shown. Predefined cutoffs and Youden-based optimal thresholds are indicated, with corresponding sensitivity and specificity values.
