# Supplementary figures and images for: Evaluation of dnDSA risk stratification using the updated PIRCHE-T2 model in two kidney transplant cohorts
Source: Front Immunol. 2026 Apr 20;17:1809989. doi: 10.3389/fimmu.2026.1809989 (PMC13135921; doi:10.3389/fimmu.2026.1809989)

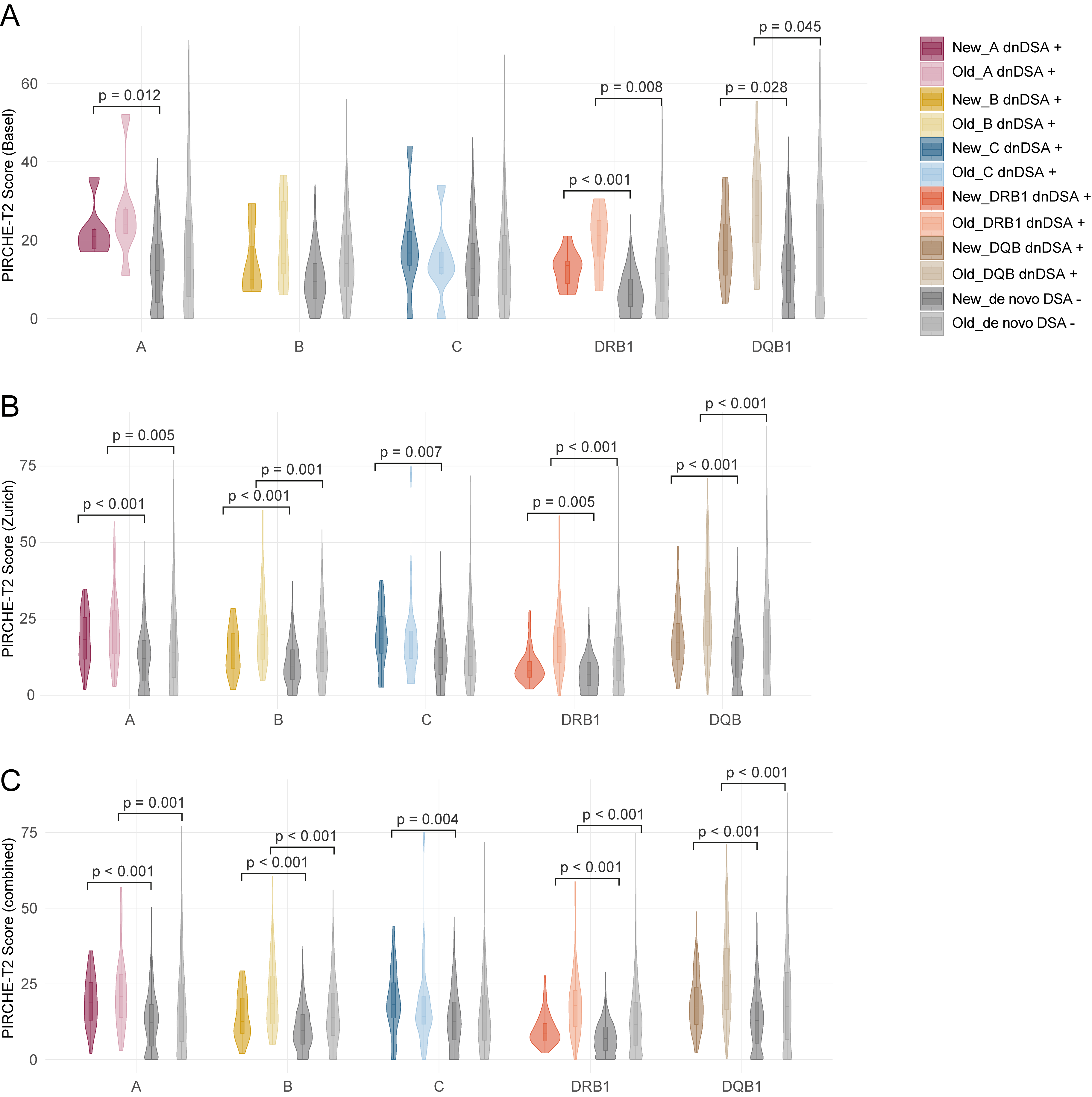

Supplement: Supplementary Figure 1 — Locus-specific PIRCHE-T2 scores stratified by dnDSA status. (A–C) Violin plots comparing PIRCHE-T2 scores between dnDSA-positive and -negative patients across individual HLA loci (HLA-A, -B, -C, -DRB1, and -DQB1) within the Basel (A), Zurich (B), and combined (C) cohorts. Each panel displays results for both the original (light color) and updated (dark color) PIRCHE-T2 models, illustrating locus-specific differences in predicted immunogenicity by dnDSA status. [file Image1.tif]

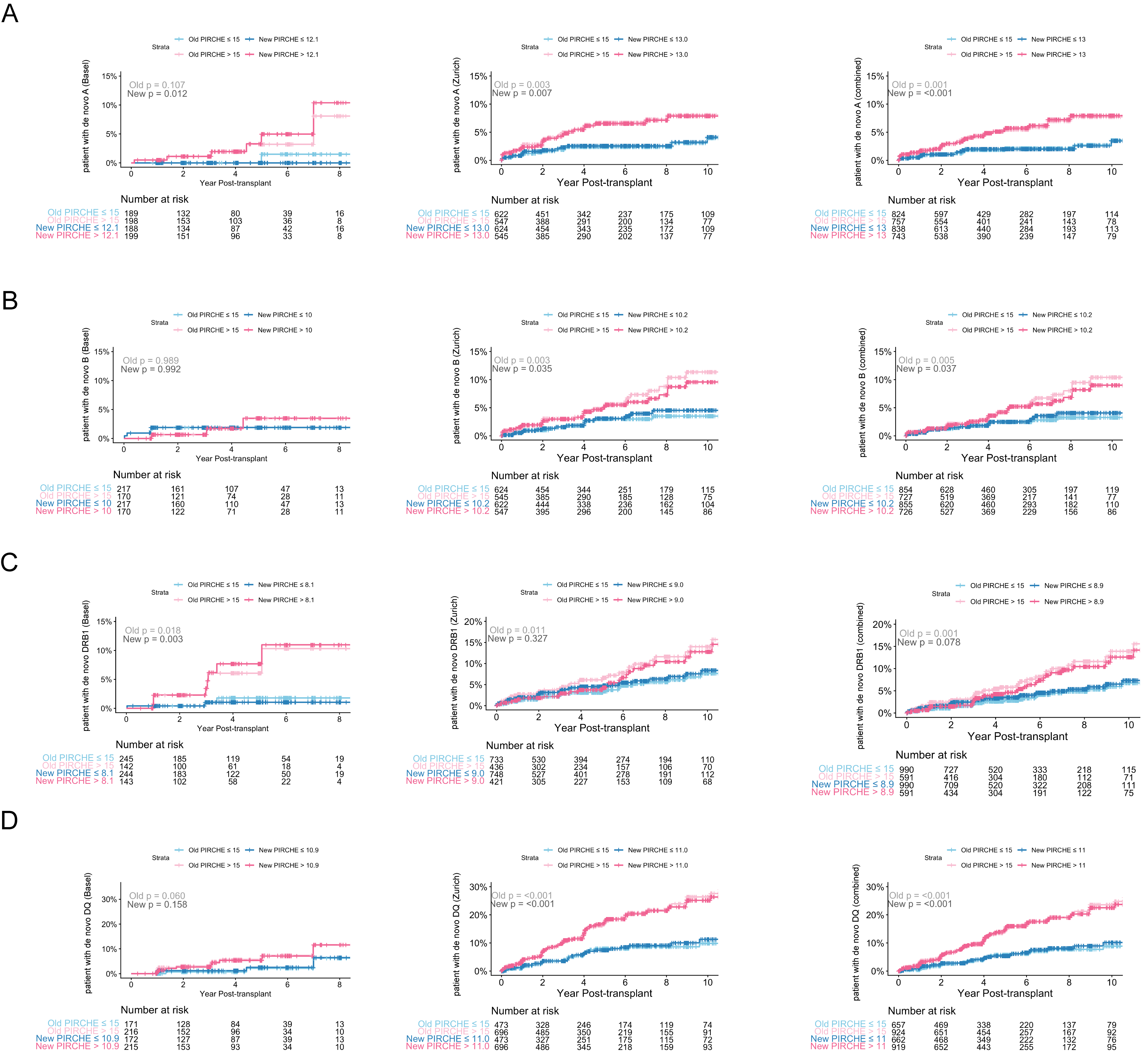

Supplement: Supplementary Figure 2 — Kaplan–Meier curves for locus-specific dnDSA development using binary PIRCHE-T2 score thresholds. (A–D) Cumulative incidence plots of dnDSA development against HLA-A, -B, -DRB1, and -DQ, respectively, across the Basel (left), Zurich (middle), and combined cohorts (right). For each locus, patients were stratified using binary PIRCHE-T2 score thresholds (original version: ≤15 vs. >15; updated version: adjusted thresholds to match patient group sizes). Both the original (light lines) and updated (dark lines) scores are shown to compare time-dependent dnDSA risk across model versions. [file Image2.tif]

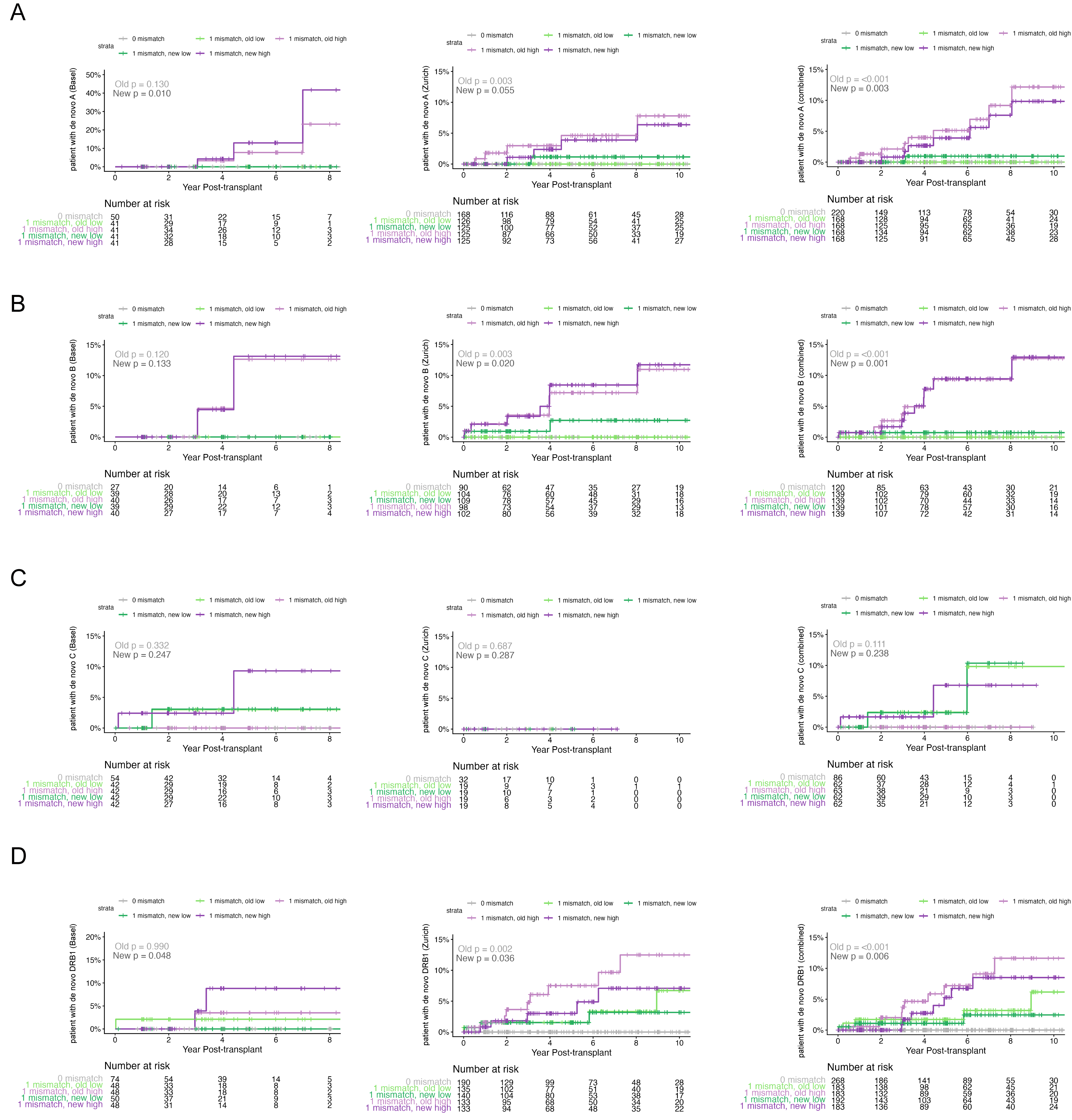

Supplement: Supplementary Figure 3 — Kaplan–Meier curves for dnDSA development in patients with one HLA mismatch per locus. (A–D) Cumulative incidence plots of dnDSA development in patients with exactly one mismatch at HLA-A, -B, -C, and -DRB1, respectively, across the Basel (left), Zurich (middle), and combined cohorts (right). Patients were divided using quantile-based thresholds of the old (light lines) and updated (dark lines) PIRCHE-T2 scores, grouped into low (bottom quantile) and high (top quantile) score categories. [file Image3.tif]

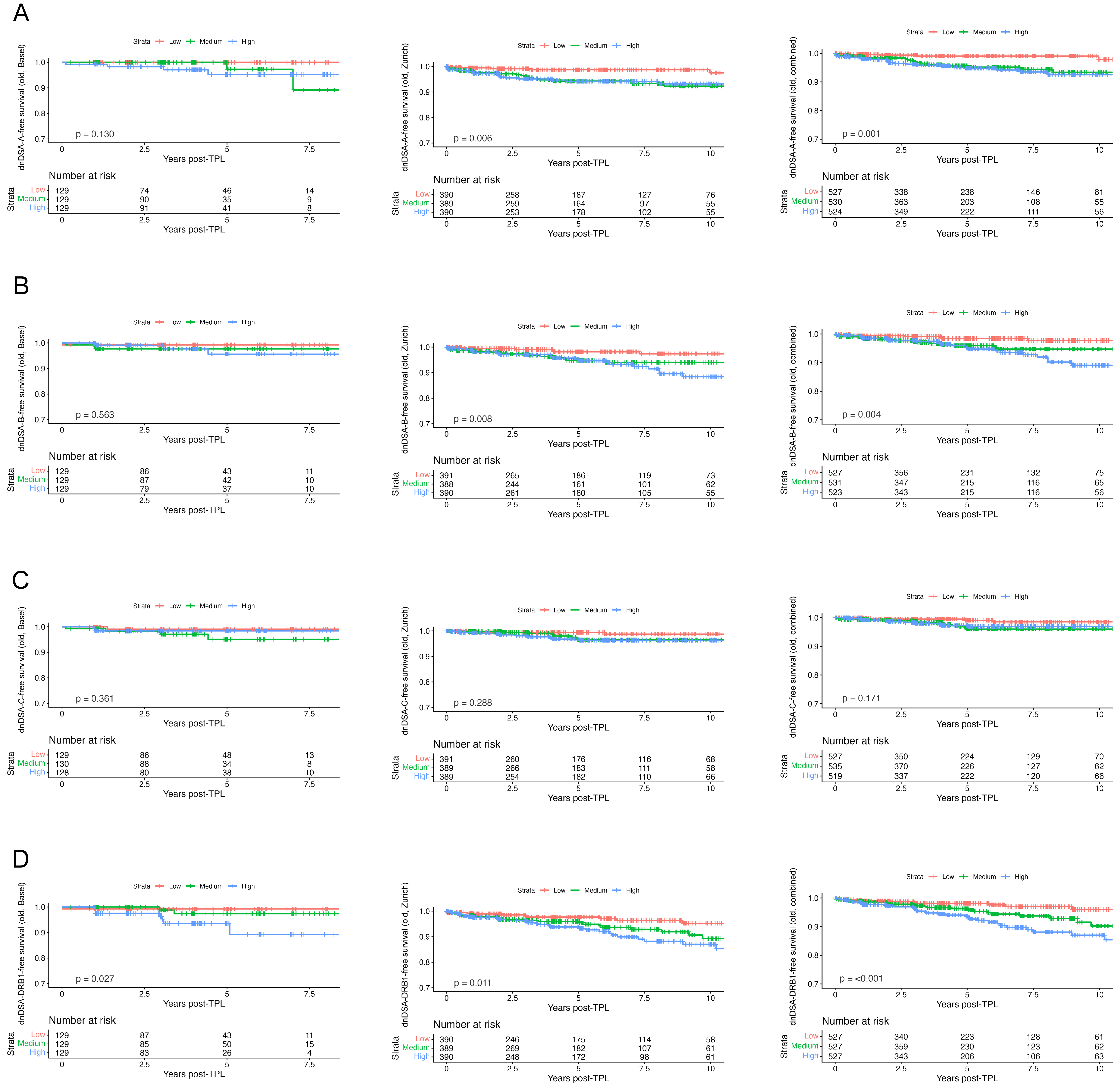

Supplement: Supplementary Figure 4 — Cox proportional hazards models for dnDSA-free survival using old PIRCHE-T2 scores. (A–E) Cox regression plots showing dnDSA-free survival stratified by tertiles of the old PIRCHE-T2 scores for HLA-A, -B, -C, -DRB1, and -DQB1. Separate analyzes are shown for the Basel (left), Zurich (middle), and combined cohorts (right). Tertile thresholds were derived from the respective score distributions within each cohort. [file Image4.tif]

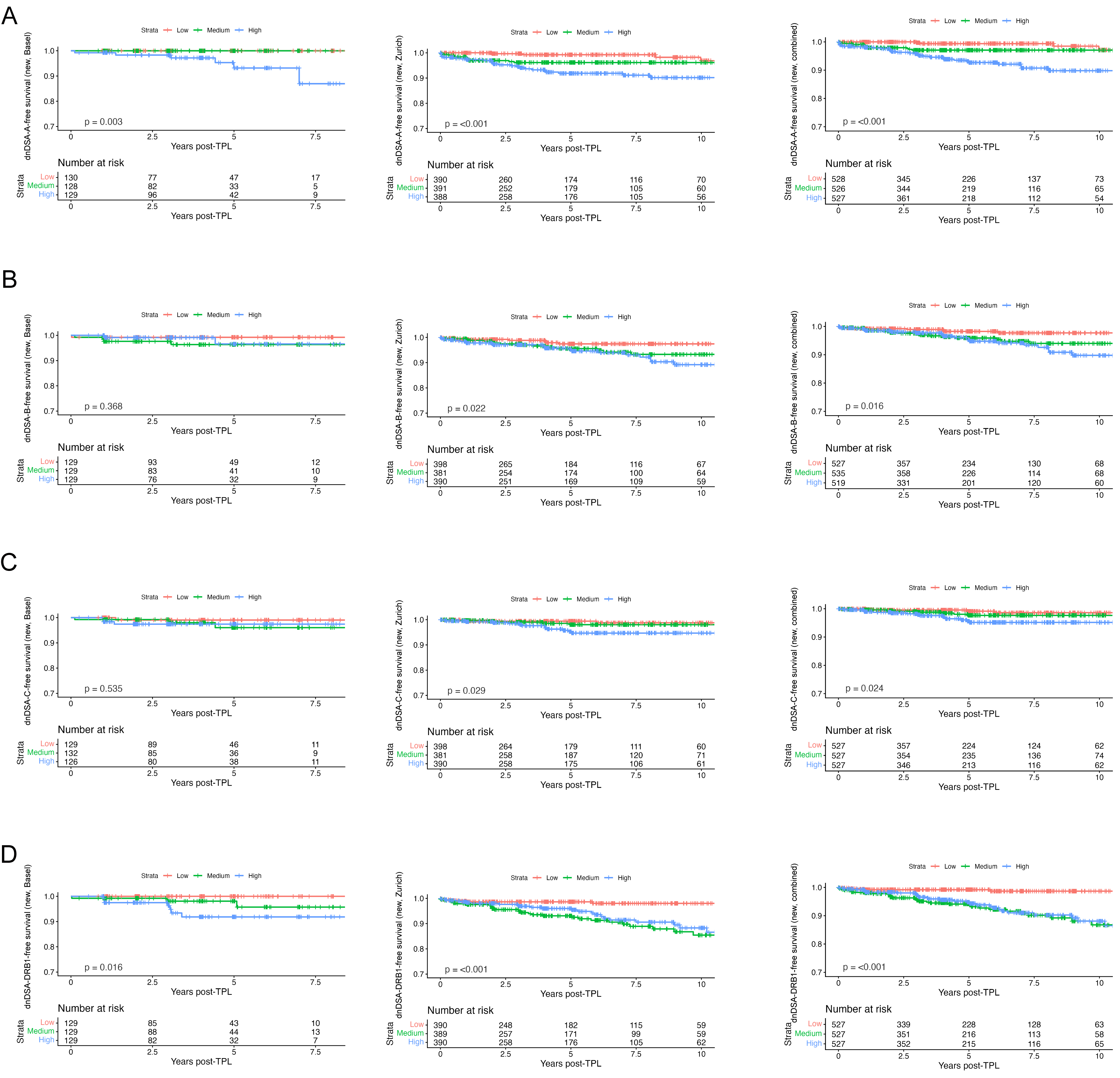

Supplement: Supplementary Figure 5 — Cox proportional hazards models for dnDSA-free survival using updated PIRCHE-T2 scores. (A–E) Cox regression plots showing dnDSA-free time stratified by tertiles of the updated PIRCHE-T2 scores for HLA-A, -B, -C, -DRB1, and -DQB1. Separate panels represent the Basel, Zurich, and combined cohorts. These curves correspond to the analyzes in Supplementary Figure 4 but use the new PIRCHE-T2 scoring model to assess changes in time-dependent predictive value. [file Image5.tif]

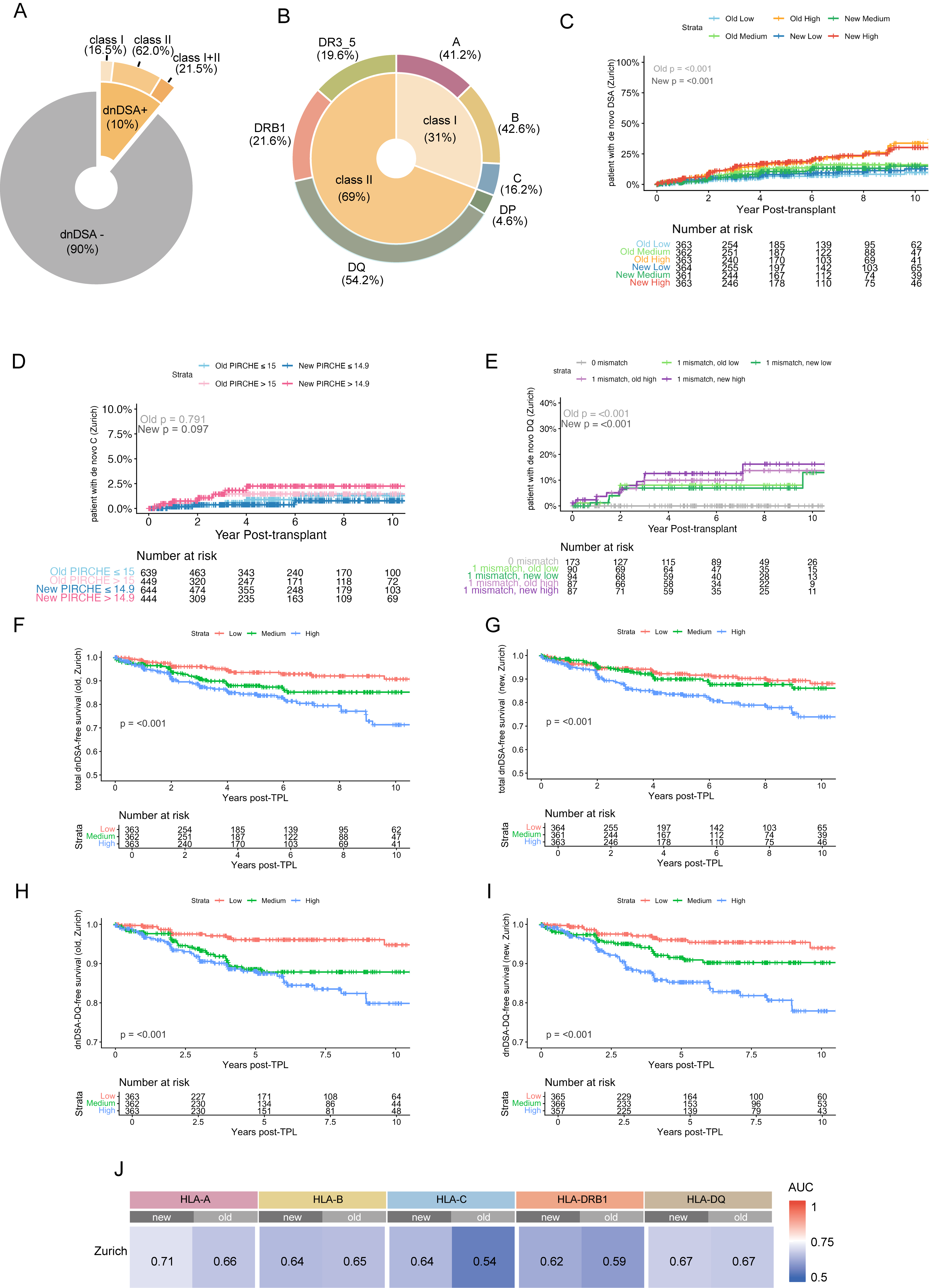

Supplement: Supplementary Figure 6 — Analyses restricted to “True” dnDSA cases in the Zurich cohort. (A) Proportion of dnDSA-positive patients considering only “True” dnDSA cases. (B) Distribution of dnDSA targets across HLA loci, grouped into class I and class II responses. (C) Cumulative incidence of dnDSA based on tertiles (low, medium, high) of total PIRCHE-T2 scores. (D) Cumulative incidence of HLA-C specific dnDSA stratified by PIRCHE-T2 score using binary thresholds. (E) Cumulative incidence of dnDSA in patients with one HLA-DQ mismatch, stratified by DQ PIRCHE-T2 score using top vs. bottom quartiles. (F–G) Cox proportional hazards models of dnDSA-free survival stratified by tertiles of total PIRCHE-T2 scores. (F) Old PIRCHE scores; (G) updated PIRCHE scores. (H–I) Cox proportional hazards models of dnDSA-free survival stratified by tertiles of HLA-DQ-specific PIRCHE-T2 scores. (H) Old PIRCHE scores; (I) updated PIRCHE scores. [file Image6.tif]

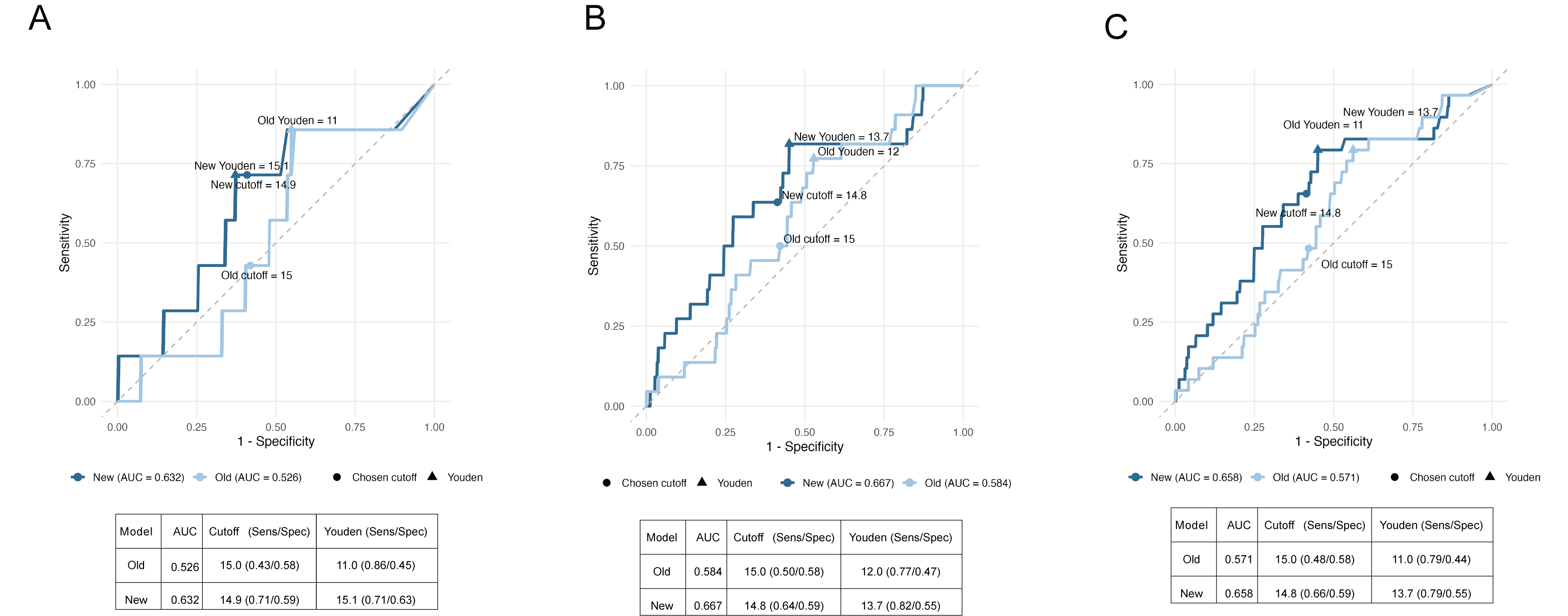

Supplement: Supplementary Figure 7 — ROC analysis of HLA-C–specific PIRCHE-T2 scores. (A–C) ROC curves for HLA-C specific PIRCHE-T2 scores in the Basel, Zurich, and combined cohorts. The original (light blue) and updated (dark blue) models are shown. Predefined cutoffs and Youden-based optimal thresholds are indicated, with corresponding sensitivity and specificity values. [file Image7.tif]
